# Gallic Acid and Taurine Attenuate Thiamethoxam-Induced Hepatotoxicity in Rats by Modulating SIRT-1/PGC-1α, NF-κB/iNOS, and p53/Bax/Caspase-3 Pathways

**DOI:** 10.3390/ph18081112

**Published:** 2025-07-25

**Authors:** Sara T. Elazab, Fatmah A. Safhi, Rasha K. Al-Akeel, Raghda H. Deraz, Souvarish Sarkar, Rania Essam Ali Gamal Eldin

**Affiliations:** 1 Department of Pharmacology, Faculty of Veterinary Medicine, Mansoura University, Mansoura 35516, Egypt; 2 Department of Biology, College of Science, Princess Nourah bint Abdulrahman University, P.O. Box 84428, Riyadh 11671, Saudi Arabia; faalsafhi@pnu.edu.sa; 3 Department of Zoology, College of Science, King Saud University, P.O. Box 2455, Riyadh 11451, Saudi Arabia; ralogial@ksu.edu.sa; 4 Department of Forensic Medicine and Clinical Toxicology, Faculty of Medicine, Zagazig University, Zagazig 44519, Egypt; raghdahamed@zu.edu.eg; 5 Department of Environmental Medicine, University of Rochester School of Medicine and Dentistry, Rochester, NY 14642, USA; rakras26@gmail.com; 6 Department of Clinical and Chemical Pathology, Faculty of Medicine, Cairo University, Giza 12613, Egypt; rania-essam@hotmail.com

**Keywords:** thiamethoxam, taurine, gallic acid, hepatic damage, oxidative stress, inflammation, apoptosis

## Abstract

**Background/Objectives**: Thiamethoxam (TMX) is one of the most extensively utilized insecticides of the neonicotinoid family; however, its application is associated with notable toxic effects on multiple organs of mammals. Our purpose was to explore the potential hepatoprotective effect of taurine (TAU) and/or gallic acid (GA) against TMX-induced liver damage, with an emphasis on their role in regulating SIRT-1/PGC-1α, NF-κB/iNOS, and p53/Bax/caspase-3 pathways. **Methods**: Rats were assigned to seven groups (*n* = 6) and gavaged daily for 28 days with saline (control group), TAU at 50 mg/kg, GA at 20 mg/kg, TMX at 78.15 mg/kg, TMX + TAU, TMX + GA, and TMX + TAU + GA. **Results**: The findings revealed that TAU and/or GA attenuated TMX-induced liver injury, as demonstrated by the restoration of hepatic performance hallmarks and histological structure. TAU and GA mitigated TMX-mediated oxidative stress and boosted the antioxidant defense mechanism by upregulating the transcription levels of SIRT-1, PGC-1α, Nrf2, and HO-1. Moreover, TAU and GA suppressed TMX-associated inflammatory response by increasing IL-10 concentration and lowering the levels of NF-κB, IL-1β, and iNOS; the mRNA levels of NLRP3; and TNF-α immunoexpression. Both compounds, individually or concurrently, exerted an anti-apoptotic effect in TMX-treated rats, evidenced by increased Bcl-2 expression and reduced p53 mRNA level, Bax expression, and caspase-3 concentration. **Conclusions**: TAU and/or GA may be regarded as promising remedies that can alleviate TMX-induced hepatotoxicity by activating SIRT-1/PGC-1α signaling and abolishing inflammation and apoptosis.

## 1. Introduction

Thiamethoxam (TMX) is a member of neonicotinoids, a widely distributed family of pesticides representing approximately 30% of globally marketed insecticides, and it is authorized in more than 120 countries across the world [1]. TMX is utilized in agricultural fields to safeguard various crops from pests such as beetles, aphids, and thrips [2]. In addition, it is one of the most commonly employed insecticides in livestock and poultry farms [3]. It achieves its insecticidal effect by targeting nicotinic acetylcholine receptors (nAChRs). Although TMX has a greater selectivity for insect nAChRs than those of mammals, its metabolites display a high affinity for mammalian nAChRs [4]. The extensive use of TMX, its persistence in crops, and its prolonged half-life in soil (>350 days) contribute to excessive human and organismal exposure through multiple pathways [5]. Moreover, Pietrzak et al. [6] elucidated that TMX, as a highly soluble neonicotinoid, is characterized by a noticeable leaching capacity. Consequently, it can readily diffuse into other ecological compartments, particularly surface water [7]. Pietrzak et al. [6] revealed that TMX levels measured in aquatic environment exceed the limit established by EU regulations. A statistical evaluation demonstrated that TMX concentrations in worldwide surface water ranged from 0.001 to 225 µg/L [8]. The Acceptable Daily Intake (ADI) for TMX in humans is set at 0.026 mg/kg/day by the European Union [9]. According to a World Health Organization (WHO) report, TMX is considered to be a class III carcinogen that mostly affects the liver and kidneys. Prior studies have reported numerous toxic effects of TMX, such as nephrotoxicity in humans and rats [10,11], hepatotoxicity in rats and rabbits [12,13,14], neurotoxicity in rats and zebrafish larvae [15,16], and reproductive toxicity in rats [17].

The liver is considered to be more prone to TMX poisoning due to its vital role in the metabolism of TMX. It has been reported that TMX causes a hepatotoxic effect in rabbits by inducing oxidative insult, releasing pro-inflammatory mediators, and modulating apoptotic signaling [14]. Therefore, alleviating the inflammatory process and oxidative insult might be an effective approach to suppress TMX hepatotoxicity. The stimulation of Sirtuin-1 (SIRT-1) has been proven to exhibit hepatoprotective effects by attenuating oxidative damage and inflammation induced by hepatotoxic xenobiotics [18,19]. It has been documented that the SIRT-1 protein is essential for numerous biological processes, including mitochondrial biogenesis, metabolism, inflammatory response, oxidative stress, the survival of cells, and apoptosis [19,20]. SIRT-1 also contributes to liver metabolism by deacetylating vital metabolic regulators such as peroxisome proliferator-activated receptor (PPAR) gamma coactivator 1 alpha (PGC-1α), which is a key modulator of oxidative metabolism and promoter of antioxidant enzymes, including catalase and manganese superoxide dismutase (MnSOD) [21,22]. In addition, SIRT-1 plays a significant role in regulating inflammatory reactions, either by deacetylating nuclear factor kappa B (NF-κB)—a transcription factor that modulates liver inflammation by managing the expression of pro-inflammatory cytokines, including tumor necrosis factor alpha (TNF-α), interleukin-1β (IL-1β), and inducible nitric oxide synthase (iNOS)—or by activating antioxidant systems to hinder free radical-induced NF-κB nuclear translocation [23]. Strong evidence suggests that enhancing SIRT-1/PGC-1α signaling can alleviate hepatic damage by stimulating mitochondrial biogenesis and mitigating oxidative insult [24,25]. Hence, the activation of this pathway can prevent TMX-induced hepatotoxicity by attenuating oxidative damage and inflammation.

Nowadays, antioxidants are receiving increased attention in the therapy of various oxidative stress-associated diseases, in addition to being considered as potential remedies for numerous disorders [26]. Gallic acid (GA) is one of the most extensively studied antioxidants. It is a naturally existing polyhydroxyphenolic compound present in several fruits, including mango, grape, pineapple, lemon, walnut, banana, and strawberry, as well as many plants. It is also found in processed beverages such as red wine and green tea [27,28]. GA exhibits a wide range of biological actions, including antioxidant, anti-inflammatory, antifungal, antiviral, anti-allergic, anticancer, and anti-mutagenic features [29,30,31]. GA has been demonstrated to afford protection against hepatotoxicity induced by doxorubicin [31], diclofenac acid [32], and thioacetamide [33], as well as against bisphenol-induced nephrotoxicity [34] and lipopolysaccharide-induced renal injury by activating SIRT-1 signaling [35].

Taurine (2-aminoethanesulfonic acid; TAU) is a free intracellular amino acid present in the heart, kidney, liver, brain, retina, and skeletal muscle of mammals, and it participates in multiple physiological procedures encompassing stabilization of cell membrane, osmoregulation, antioxidant defense, calcium homeostasis, bile acid conjugation, and detoxification [36]. It is a byproduct of L-cysteine breakdown that can promote reduced glutathione (GSH) concentrations inside the cells [37]. Ripps and Shen [38] indicated that TAU can quench the hydroxyl radicals and alleviate the damaging effects of oxygen radicals. It has been reported that TAU displays an ameliorative action against hepatotoxicity caused by carbon tetrachloride [39], cyclosporine A [40], malathion [41], and doxorubicin [42]. Liu et al. [43] stated that TAU showed palliative action against cardiac disorders caused by pressure overload through alleviating oxidative stress and apoptosis of cardiomyocytes by stimulating SIRT-1 and repressing p53.

So far as the authors are aware, the role of GA and TAU, whether administered individually or in combination, in mitigating TMX-induced liver toxicity has not yet been investigated. Therefore, the present research was designed to assess the potential ameliorative effects of individual and/or combined treatment with GA and TAU on TMX-induced hepatic injury and to elucidate the involved mechanisms, with a particular focus on the SIRT-1/PGC-1α, NF-κB/iNOS, and p53/Bcl-2-associated X protein (Bax)/caspase-3 signaling pathways. This study aimed to explore the crosstalk and mechanistic interplay among these key pathways through integrated biochemical, molecular, and histological analyses to provide a comprehensive understanding of the palliative potential of GA and TAU against TMX-induced hepatotoxicity.

## 2. Results

### 2.1. TAU and/or GA Improve the Body Weight and Hepatosomatic Index in TMX-Exposed Rats

Rats exposed to TMX alone displayed a remarkable decline (*p* < 0.01) in final body weight (FBW) (recorded on the 28th day of the trial) and a significant increase (*p* < 0.001) in the hepatosomatic index relative to the controls. On the contrary, FBWs were significantly increased (*p* < 0.05) in rats that received either TAU or GA alongside TMX versus the TMX group. It is worth mentioning that treatment of TMX-exposed rats with a combination of TAU and GA revealed noticeable improvement in percentage change in weight, FBW, and hepatosomatic index compared to individual therapy with either one of them (Table 1 and Supplementary Table S1).

### 2.2. TAU and/or GA Mitigate Hepatic Impairment Caused by TMX

The results revealed a marked increase (*p* < 0.001) in ALT and ALP activities, as well as in total bilirubin levels, accompanied by a drastic drop (*p* < 0.001) in the concentrations of albumin and total protein in rats that received TMX alone compared to the controls. In contrast, rats that ingested TAU or GA with TMX showed a remarkable reduction (*p* < 0.001) in total bilirubin levels and ALT and ALP activities, along with a considerable elevation in albumin (*p* < 0.05 and *p* < 0.01, respectively) and total protein levels (*p* < 0.01 and *p* < 0.001, respectively) with respect to those exposed to TMX only. Notably, co-administration of TAU and GA in TMX-exposed rats normalized these liver function parameters (Table 2).

### 2.3. TAU and/or GA Ameliorate TMX-Induced Oxidative Insult in Liver Tissues

As illustrated in Table 3, exposure to TMX caused a noticeable increase (*p* < 0.001) in hepatic MDA and NO contents, accompanied by a marked drop (*p* < 0.001) in liver GSH content and the hepatic activities of SOD and CAT relative to the controls. In contrast, the TMX + TAU and TMX + GA groups displayed a dramatic decrease in the concentrations of MDA (*p* < 0.05 and *p* < 0.001, respectively) and NO (*p* < 0.01 and *p* < 0.001, respectively), along with a notable increase in the activities of SOD (*p* < 0.05), CAT (*p* < 0.05 and *p* < 0.001, respectively), and GSH concentrations (*p* < 0.05 and *p* < 0.001, respectively) as compared to the TMX group. Moreover, administration of TAU combined with GA to TMX-treated rats revealed the best palliative action on these redox status indices, where these parameters were restored to their control levels.

### 2.4. TAU and/or GA Modulate the Hepatic Concentrations of SIRT-1, PGC-1α, NF-κB, Phospho-NF-κB p65, iNOS, IL-1β, IL-10, p53, Caspase-3, and Cleaved Caspase-3 in TMX-Exposed Rats

**Figure 1:**
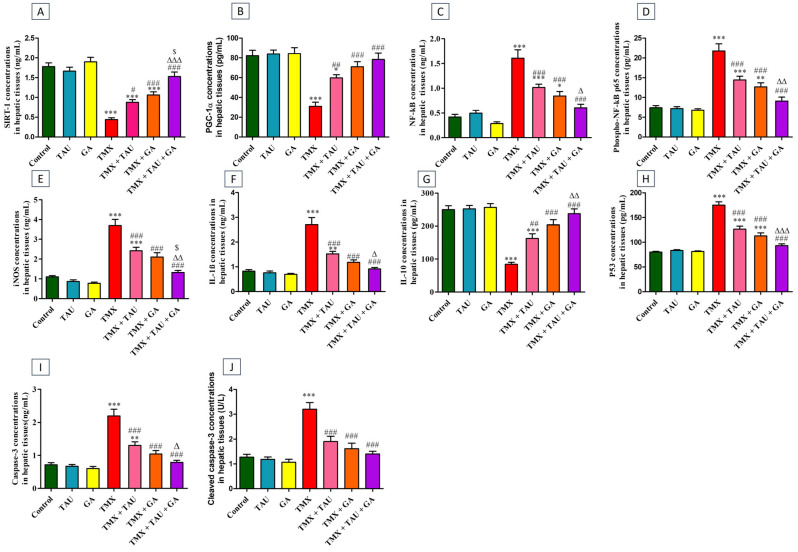
Effect of taurine and/or gallic acid on the concentrations of SIRT-1 (**A**), PGC-1α (**B**), NF-κB (**C**), phospho NF-κB p65 (**D**), iNOS (**E**), IL-1β (**F**), IL-10 (**G**), p53 (**H**), caspase-3 (**I**), and cleaved caspase-3 (**J**) in the hepatic tissues of rats exposed to TMX. Results are displayed as mean ± SEM (n = 6). One-way ANOVA followed by Tukey’s post hoc test was applied for statistical comparison. * *p* < 0.05, ** *p* < 0.01, and *** *p* < 0.001 vs. the control group; ^#^ *p* < 0.05, ^##^ *p* < 0.01 and ^###^ *p* < 0.001 vs. the TMX group; ^∆^ *p* < 0.05, ^∆∆^ *p* < 0.01, and ^∆∆∆^ *p* < 0.001 vs. the TMX + TAU group; and ^$^ *p* < 0.05 vs. the TMX + GA group. TAU, taurine; GA, gallic acid; TMX, thiamethoxam; SIRT-1, Sirtuin-1; PGC-1α, peroxisome proliferator-activated receptor (PPAR) gamma coactivator 1 alpha; NF-κB, nuclear factor kappa B; phospho NF-κB p65, phosphorylated NF-κB p65; iNOS, inducible nitric oxide synthase; IL-1β, interleukin-1β; IL-10, interleukin-10.

Compared to control rats, the TMX group exhibited a remarkable increase (*p* < 0.001) in the liver concentrations of NF-κB (Figure 1C), phosphorylated NF-κB p65 (phospho-NF-κB p65, Figure 1D), iNOS (Figure 1E), IL-1β (Figure 1F), p53 (Figure 1H), caspase-3 (Figure 1I), and cleaved caspase-3 (Figure 1J), together with a marked decline (*p* < 0.001) in the levels of SIRT-1 (Figure 1A), PGC-1α (Figure 1B), IL-10 (Figure 1G). In contrast, a significant decrease (*p* < 0.001) in the levels of NF-κB, phospho-NF-κB p65, iNOS, IL-1β, p53, caspase-3, and cleaved caspase-3 and a noticeable increase in SIRT-1, PGC-1α, and IL-10 levels were observed in TMX-exposed rats that were gavaged with either TAU (*p* < 0.05, *p* < 0.01, *p* < 0.01, respectively) or GA (*p* < 0.001) relative to those exposed to TMX only. The expression levels of these proteins reverted back to their basal levels in the TMX + TAU + GA group.

### 2.5. TAU and/or GA Regulate the Transcription Levels of SIRT-1, PGC-1α, Nrf2, HO-1, NLRP3, and p53 in Liver Tissues of TMX-Administered Rats

**Figure 2:**
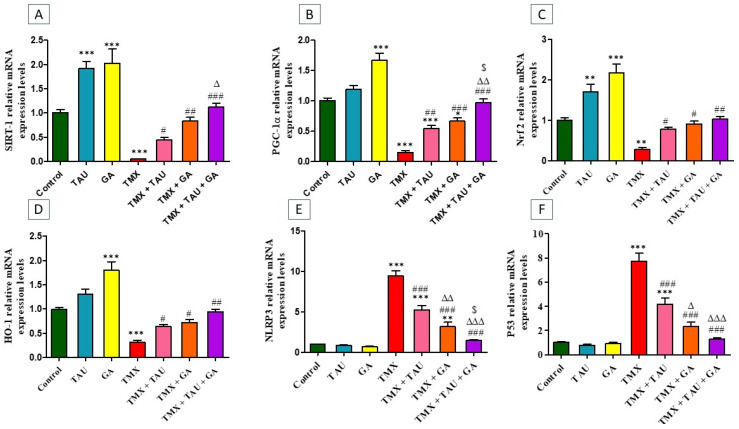
Impact of taurine and/or gallic acid on the transcription levels of SIRT-1 (**A**), PGC-1α (**B**), Nrf2 (**C**), HO-1 (**D**), NLRP3 (**E**), and p53 (**F**) in liver tissues of rats that received TMX for 4 weeks. Values are demonstrated as mean ± SEM (n = 6). The findings of the tested groups were compared using one-way ANOVA followed by Tukey’s post hoc analysis.* *p* < 0.05, ** *p* < 0.01, and *** *p* < 0.001 vs. the control group; ^#^ *p* < 0.05, ^##^ *p* < 0.01, and ^###^ *p* < 0.001 vs. the TMX group; ^∆^ *p* < 0.05, ^∆∆^ *p* < 0.01, ^∆∆∆^ *p* < 0.001 vs. the TMX + TAU group; and ^$^ *p* < 0.05 vs. the TMX + GA group. TAU, taurine; GA, gallic acid; TMX, thiamethoxam; SIRT-1, Sirtuin-1; PGC-1α, peroxisome proliferator-activated receptor (PPAR) gamma coactivator 1 alpha; Nrf2, nuclear factor erythroid 2-related factor-2; HO-1, heme oxygenase-1; NLRP3, nucleotide-binding domain, leucine-rich-containing family, pyrin domain-containing-3.

TMX-treated rats exhibited a statistical downregulation in the transcription levels of SIRT-1 (*p* < 0.001, Figure 2A), PGC-1α (*p* < 0.001, Figure 2B), Nrf2 (*p* < 0.01, Figure 2C), and HO-1 (*p* < 0.001, Figure 2D) genes, besides a marked upregulation (*p* < 0.001) in the mRNA expressions of NLRP3 and p53, relative to the control ones. Conversely, providing the poisoned rats with either TAU or GA alleviated TMX-induced disorders in the expression of these genes. Notably, a combination remedy of TAU and GA in poisoned rats resulted in complete restoration of the transcription levels of all assessed genes.

### 2.6. Multivariate Analysis

**Figure 3:**
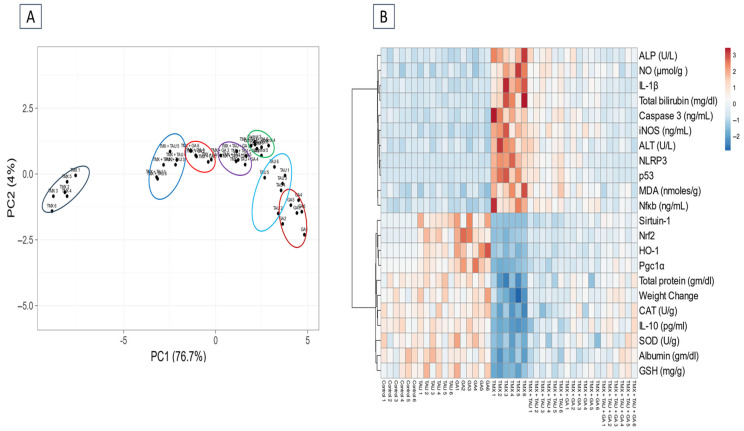
Multivariate analysis of entire datasets from serum and hepatic tissues of TMX-exposed rats upon TAU and/or GA intervention. (**A**) Plot of principal component analysis (PCA) for identifying all trial groups. (**B**) Clustering heat map. The horizontal axis represents various study groups, while the vertical axis points out the variables compared in groups. Each row exhibits the variable values in different rat samples, and each column represents all variable values in each rat sample. Every cell’s color in the map indicates a variable value. Blue color has the lowest value on the grading scale, whereas red color has the highest value. TAU, taurine; GA, gallic acid; TMX, thiamethoxam; Control1-6, samples of control groups; TAU1-6, samples of TAU group; GA1-6, samples of the GA group; TMX + TAU1-6, samples of the TMX + TAU group; TMX + GA1-6, samples of TMX + GA; TMX + TAU + GA1-6, samples of the TMX + TAU+ GA group.

As delineated in Figure 3A, PCA was applied to assess the correlation between various interventions and covariates. PCA revealed that TMX-administered rats clustered together and were clearly distinct from other treated rats. The rats that received TMX + TAU + GA clustered closer to the controls and separated from the TMX-exposed ones. These outcomes indicated a considerable distinction between the poisoned rats with TMX and those that received TMX + TAU + GA, suggesting that the combination therapy with TAU and GA can significantly alleviate the toxic effects of TMX. Monotherapy with either TAU or GA can also attenuate TMX-induced hepatotoxicity but is not as efficacious as the combined therapy. Moreover, the clustering heat map was generated to provide an intuitive overview of the entire dataset (Figure 3B). It clarifies the remarkable distinction between the values of all variables after different interventions. These data illustrate that TMX-treated rats exhibited more damage than rats in other groups. Co-administration of TAU and GA alongside TMX led to a phenotype similar to controls, while individual therapy with either TAU or GA treatment led to a phenotype in between the controls and TMX-intoxicated rats, suggesting a partial rescue.

### 2.7. TAU and/or GA Alleviate TMX-Induced Hepatic Histological Lesions

**Figure 4:**
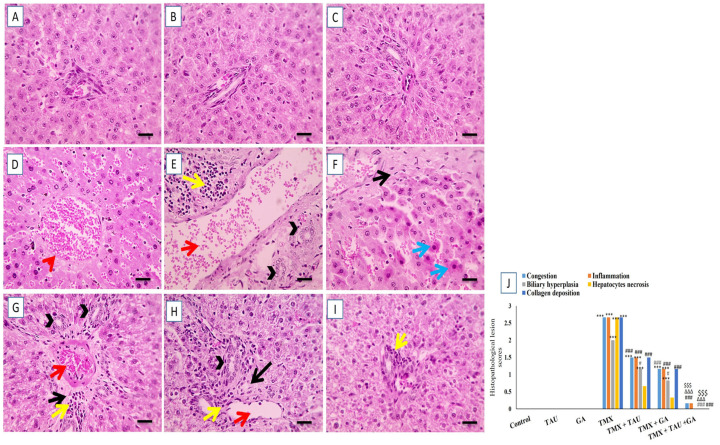
Photomicrographs of hepatic slides from rats prepared with H&E stain. (**A**) Control, (**B**) taurine, and (**C**) gallic acid groups: revealing normal well-arranged hepatic cords surrounding typical portal areas and normal sinusoids. (**D**–**F**) Thiamethoxam (TMX) group: exhibiting congested central veins (red arrowheads), infiltration of leukocytes (yellow arrows), widening of portal areas as a result of portal vein congestion (red arrows), biliary hyperplasia (black arrowheads), hepatocytes necrosis (blue arrows), and excess collagen deposition (black arrows). (**G**) TMX + TAU: displaying slightly decreased expansion of portal areas (red arrows), reduced collagen deposition (black arrows), less leukocytic cell infiltration (yellow arrows), and biliary hyperplasia (black arrowheads). (**H**) TMX + GA: demonstrating moderately reduced expansion of portal areas (red arrows), few leukocytic cell infiltration (yellow arrows), collagen deposition (black arrows), and biliary hyperplasia (black arrowheads). (**I**) TMX + TAU + GA: revealing noticeable decrease in the lesions with very little leukocytic cell infiltration (yellow arrows). (**J**) The scoring of the observed lesions in hepatic sections. One-way ANOVA followed by Tukey’s test was utilized to compare the obtained data. *** *p* < 0.001 vs. the control group; ^#^ *p* < 0.05 and ^###^ *p* < 0.001 vs. the TMX group. ^∆∆∆^ *p* < 0.001 vs. the TMX + TAU group; ^$$$^ *p* < 0.001 vs. the TMX + GA group. X400 bar, 50 μm.

The microscopic screening of the prepared hepatic slides demonstrated normal, well-organized hepatic cords around standard portal areas with typical sinusoids in the control, TAU, and GA groups (Figure 4A–C). In contrast, slides from rats in the TMX group revealed congested central veins, necrosis of hepatocytes, expansion of portal areas as a sequela of congested portal veins, excessive buildup of collagen, leukocytic cell infiltration, and biliary hyperplasia (Figure 4D–F). Sections from rats in the TMX + TAU group exhibited a slight reduction in the expansion of portal regions, along with decreased collagen deposition, less leukocytic cell infiltration, and biliary hyperplasia (Figure 4G). Moreover, TMX + GA-treated rats displayed a moderate decrease in the expansion of portal areas, along with reductions in leukocytic cell infiltration, collagen deposition, and biliary hyperplasia (Figure 4H). Additionally, a marked reduction in the lesions, with very little leukocytic cell infiltration, was recorded in liver specimens of the TMX + TAU + GA group (Figure 4I). The scoring of the microscopic structural lesions in liver sections is depicted in Figure 4J.

**Figure 5:**
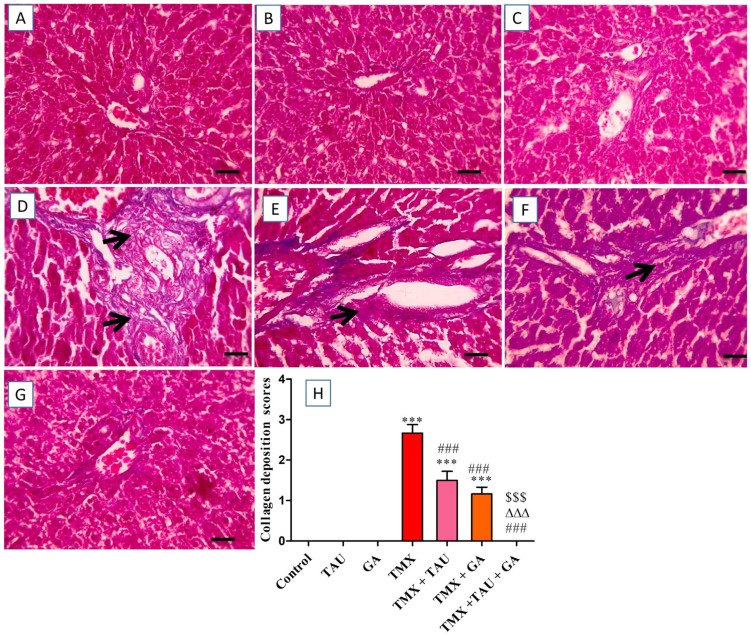
Microscopic pictures of liver sections stained with Masson’s trichrome. (**A**) Control, (**B**) taurine, and (**C**) gallic acid groups: indicating no collagen deposition in portal regions. (**D**) Thiamethoxam (TMX) group: denoting excessive bluish collagen deposition (black arrows) in portal zones. (**E**) TMX + TAU: showing moderate bluish collagen deposition (black arrows) in portal areas. (**F**) TMX + GA: clarifying mild bluish collagen deposition (black arrows) in portal areas. (**G**) TMX + TAU + GA: exhibiting no deposition of collagen fibers in portal regions. (**H**) This reveals collagen deposition scores in liver slides from the study groups. The scores are presented as mean ± SEM. The scores of collagen depositions were compared between the trial groups employing one-way ANOVA and Tukey’s post hoc test. *** *p* < 0.001 vs. the control group. ^###^ *p* < 0.001 vs. the TMX group. ^∆∆∆^ *p* < 0.001 vs. the TMX + TAU group; and ^$$$^ *p* < 0.001 vs. the TMX + GA group. X400 bar, 50 μm.

The histological investigation of liver specimens stained with Masson’s trichrome revealed an absence of collagen deposition in portal areas in the control, TAU, and GA groups (Figure 5A–C). Conversely, liver sections from rats in the TMX group exhibited extensive bluish collagen accumulation in portal regions (Figure 5D). Meanwhile, rats treated with TMX + TAU displayed moderate bluish fibrous tissue deposition in portal zones (Figure 5E). Additionally, mild accumulation of bluish collagen in portal areas was noticed in hepatic tissues of rats in the TMX + GA group (Figure 5F). Furthermore, the liver slides from the TMX + TAU + GA group revealed no accumulation of bluish collagen in portal zones (Figure 5G). The scores of collagen deposition in the hepatic architecture of the tested groups are shown in Figure 5H.

### 2.8. TAU and/or GA Modulate the Immunoexpression of TNF-α, Bax, and Bcl-2 in the Liver of TMX-Treated Rats

**Figure 6:**
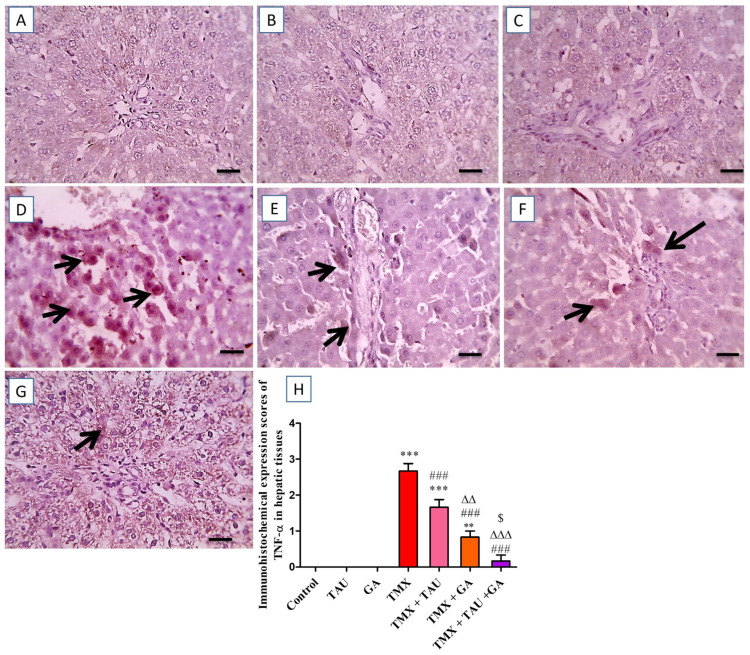
Images of immunostained hepatic sections against TNF-α. (**A**) Control, (**B**) taurine, and (**C**) gallic acid groups: displaying negative expression in hepatocytes. (**D**) Thiamethoxam (TMX) group: revealing remarkable positive brown reaction for TNF-α expression in numerous hepatic cells surrounding the affected portal regions (black arrows). (**E**) TMX + TAU: presenting slightly decreased positive brown expression of TNF-α in some hepatocytes adjacent to the affected portal areas (black arrows). (**F**) TMX + GA: showing moderate diminution in the brown expression TNF-α in fewer hepatocytes around portal regions (black arrows). (**G**) TMX + TAU + GA: representing mild brown staining for TNF-α expression in individual hepatocytes around portal zones (black arrows). (**H**) This shows the immunoexpression scores of TNF-α in liver tissues of the trial groups. Values are depicted as mean ± SEM. One-way ANOVA and Tukey’s post hoc method were applied to compare the TNF-α immunoexpression scores between the groups. ** *p* < 0.01 and *** *p* < 0.001 vs. the control group. ^###^ *p* < 0.001 vs. the TMX group; ^∆∆^ *p* < 0.01 and ^∆∆∆^ *p* < 0.001 vs. the TMX + TAU group; and ^$^ *p* < 0.05 vs. the TMX + GA group. X400 bar, 50 μm.

The hepatic sections immunostained against TNF-α demonstrated no immunoreactivity in the hepatocytes of the control, TAU, and GA groups (Figure 6A–C). In contrast, a marked positive brown immunoreactivity in many hepatocytes encircling the afflicted portal areas was identified in the tissues of the TMX group (Figure 6D). Nevertheless, rats gavaged with TAU alongside TMX displayed a slight reduction in the positive brown reaction in hepatocytes surrounding the affected portal regions (Figure 6E). Furthermore, the TMX + GA group showed moderately decreased positive immunoreactivity in fewer hepatocytes around the afflicted portal zones (Figure 6F). Additionally, the prepared section from TMX + TAU + GA rats revealed mild positive immunoexpression in individual hepatocytes adjacent to portal areas (Figure 6G). The scores of TNF-α immunohistochemical staining in all examined slides are represented in Figure 6H.

**Figure 7:**
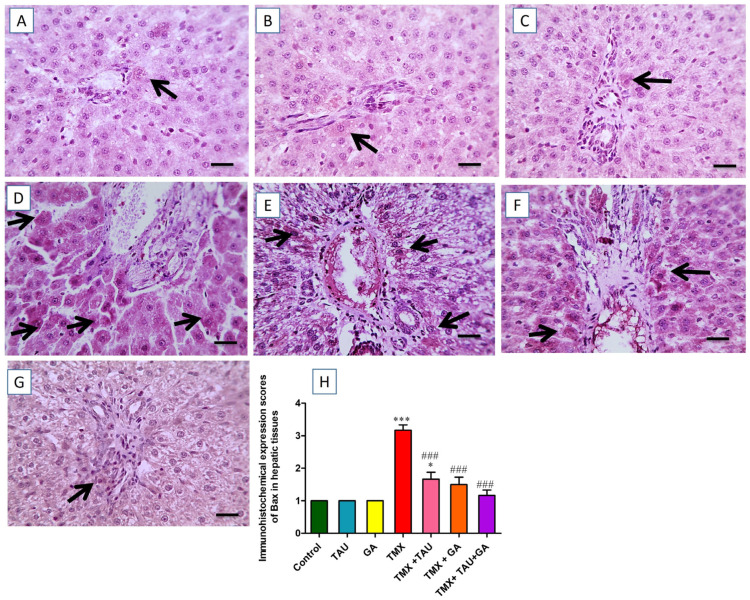
Histological screening of immunostained hepatic slides against Bax. (**A**) Control, (**B**) taurine, and (**C**) gallic acid groups: representing mild positive Bax expression in individual hepatocytes encircling normal portal areas. (**D**) Thiamethoxam (TMX) group: showing marked brown staining (positive Bax expression) in multiple hepatocytes around affected portal zones (black arrows). (**E**) TMX + TAU: denoting a slight decline in the positive brown Bax expression in some hepatocytes around affected portal areas (black arrows). (**F**) TMX + GA: exhibiting moderately decreased positive Bax reaction (brown staining) in fewer hepatic cells adjacent to the afflicted portal regions (black arrows). (**G**) TMX + TAU + GA: presenting mild brown staining for Bax expression in individual hepatic cells around mildly afflicted portal regions (black arrows). (**H**) This displays the immunohistochemical staining scores of Bax in the slides of the investigated groups. Data are delineated as mean ± SEM. The scores of Bax immunohistochemical staining were compared between groups using one-way ANOVA and Tukey’s post hoc test. * *p* < 0.05 and *** *p* < 0.001 vs. the control group; and ^###^ *p* < 0.001 vs. the TMX group. X400 bar, 50 μm.

The images captured by the ordinary microscope for the immunostained liver slices against Bax revealed mild brown immunoreactivity in individual hepatocytes surrounding normal portal regions in the control, TAU, and GA groups (Figure 7A–C). On the other hand, the TMX group exhibited intense brown immunoexpression in numerous hepatocytes neighboring the impacted portal regions (Figure 7D). Meanwhile, slightly diminished positive brown expression in some hepatocytes near the insulted portal areas was detected in the photomicrographs taken from the TMX + TAU group (Figure 7E). In addition, the slides from the TMX + GA group exhibited moderately reduced brown staining present in fewer hepatocytes near the afflicted portal areas (Figure 7F). Moreover, the TMX + TAU + GA group showed mild positive immunoexpression in individual hepatocytes encircling mildly affected portal zones (Figure 7G). The scores of Bax staining in liver tissues are illustrated in Figure 7H.

**Figure 8:**
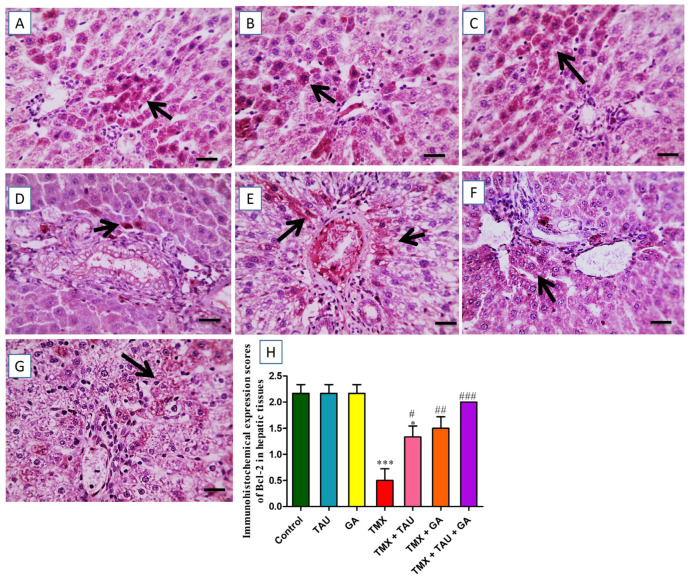
Immunohistochemical-stained photomicrographs for Bcl-2 in hepatic specimens of investigated rats. (**A**) Control, (**B**) taurine, and (**C**) gallic acid groups: showing noticeable brown Bcl-2 expression in many hepatocytes surrounding typical portal areas. (**D**) Thiamethoxam (TMX) group: exhibiting prominent reduction in positive brown Bcl-2 expression in hepatocytes encircling the afflicted portal areas (black arrows). (**E**) TMX + TAU: presenting slightly increased positive Bcl-2 expression in fewer hepatic cells surrounding affected portal areas (black arrows). (**F**) TMX + GA: clarifying moderate increase in the brown staining (Bcl-2 expression) in some hepatocytes adjacent to the afflicted portal regions (black arrows). (**G**) TMX + TAU + GA: designating remarkable positive brown staining for Bcl-2 in several hepatocytes encircling mildly affected portal zones. (**H**) This demonstrates the immunoexpression scores of Bcl-2 in hepatic tissues of all groups. The scores are shown as mean ± SEM. One-way ANOVA and Tukey’s post hoc test were implemented to compare Bcl-2 immunohistochemical scores. * *p* < 0.05 and *** *p* < 0.001 vs. the control group; ^#^ *p* < 0.05, ^##^ *p* < 0.01, and ^###^ *p* < 0.001 vs. the TMX group.X400 bar, 50 μm.

The slides immunostained against Bcl-2 showed remarkable positive brown expression in multiple hepatocytes neighboring the intact portal regions in the control, TAU, and GA groups (Figure 8A–C). In contrast, the photos from TMX-treated rats demonstrated a considerable decline in the brown staining reaction in hepatocytes encircling the insulted portal zones (Figure 8D). However, animals exposed to TMX + TAU exhibited a slight increase in positive expression in fewer hepatocytes near the impacted portal regions (Figure 8E). Furthermore, the TMX + GA group displayed moderately increased positive reaction in some hepatocytes surrounding the afflicted portal zones (Figure 8F). Rats that received a combination of TAU and GA presented noticeable brown expression in several hepatocytes near the afflicted portal areas (Figure 8G). The scores of Bcl-2 expression in the hepatic architecture of the trial groups are delineated in Figure 8H.

## 3. Discussion

The current investigation sheds light on the efficacy of GA and TAU, either individually or concomitantly, in mitigating TMX-induced hepatic injury in rats. This palliative effect is evidenced by the improvement in liver function, repair of the histological architecture, and restoration of redox homeostasis, in addition to the suppression of inflammatory response and apoptotic alterations through modulating SIRT-1/PGC-1α, NF-κB/iNOS, and p53/Bax/caspase-3 signaling pathways. GA showed a more pronounced abrogative action relative to TAU against hepatic injury induced by TMX. Notably, combined therapy with GA and TAU exhibited a more remarkable protective effect against TMX-induced hepatotoxicity than monotherapy, indicating synergistic anti-inflammatory, antioxidant, and anti-apoptotic effects of both candidates. These findings pave the way for using the combination of GA and TAU as a prospective avenue for combating TMX-induced hepatotoxicity.

In this trial, the results demonstrated that TMX ingestion by rats at a dose of 78.15 mg/kg/day (1/20 LD50) for 4 weeks led to a dramatic reduction in FBW along with noticeable compromised liver function evidenced by increased ALT and ALP activities, enhanced total bilirubin levels, and decreased serum concentrations of albumin and total protein. The increase in serum ALT activity could be attributed to TMX-induced hepatotoxicity, which disturbs the permeability of the cell membrane, resulting in leakage of lysosomal enzymes and an increase in ALT release [44,45]. According to Kaneko et al. [46], elevated ALT is a typical hallmark for hepatic injury as it reflects the alterations in cell wall permeability caused by liver insult. Similar findings were reported by Abdel-Razik et al. [11] and El-Sheikh et al. [47] in rats exposed to TMX at doses of 31.26 mg/kg and 78.15 mg/kg, respectively, daily for 28 days.

Supporting this observed hepatic malfunction, histological examination of liver slides from TMX-treated rats demonstrated disruption of hepatic architecture characterized by congested central veins, broadening of portal zones due to congestion in portal vein, excessive collagen accumulation, necrosis of hepatic cells, and infiltration of leukocytic cells. In the same vein, Abdel-Razik et al. [11] reported serious congestion of central veins, fibrosis, necrosis, and infiltration of inflammatory cells in hepatic tissues of TMX-treated rats.

The current research demonstrated that treatment with TAU and/or GA displayed promising effects in countering TMX-induced hepatotoxicity, as evidenced by alleviation of functional and biochemical disturbances along with restoration of the hepatic histological profile. These outcomes were consistent with those of Gedikli et al. [42], who reported that TAU corrected the increased ALT activity in DOX-exposed rats. Similarly, Timbrell et al. [48] explained that TAU prevents the leakage of biochemical markers through its membrane-stabilizing effects. Moreover, numerous researchers have demonstrated that TAU has a protective action on hepatic tissues against different xenobiotics [41,49,50,51]. In addition, GA has been proven to mitigate cyclophosphamide-induced disorders in hepatic performance markers, including ALT, ALP, and total bilirubin [52]. Furthermore, several prior investigators have documented the palliative action of GA against hepatic damage caused by doxorubicin [31], diclofenac [32], carbon tetrachloride [53], and fenitrothion [54].

Neonicotinoids have been reported to damage mammalian cellular components, including proteins, DNA, and lipids, by inducing oxidative stress through increasing the production of ROS and reactive nitrogen species, in addition to suppressing the activities of antioxidant enzymes [11,55]. Yan et al. [56] identified oxidative stress as the leading factor in hepatic injury caused by TMX. In the present study, TMX administration resulted in serious oxidative stress, as indicated by a substantial spike in MDA content and a dramatic reduction in SOD, CAT activities, and GSH concentration in comparison with the control rats. The decline in the activities of SOD and CAT denotes their exhaustion during the metabolic breakdown of superoxide radicals and H_2_O_2_ produced from the vigorous oxidative stress caused by TMX [57,58]. Our findings align with those of Abdel-Razik et al. [11]. Furthermore, the results showed an increase in NO levels in hepatic tissues of TMX-treated rats. This increase in NO content may be attributed to the TMX-induced activation of iNOS. Similarly, Duzguner and Erdogan [59] recorded an increase in NO levels in the brain and hepatic tissues of rats that received imidacloprid (IMI), another neonicotinoid.

TMX-mediated oxidative insult is explained by the marked suppression in the mRNA expression of SIRT-1, PGC-1α, Nrf2, and HO-1, along with reduced protein concentrations of SIRT-1 and PGC-1α. Abduh et al. [60] documented that SIRT-1/PGC-1α and NRf2/HO-1 signaling play crucial roles in regulating redox homeostasis and protecting against tissue inflammation. SIRT-1 has been recognized to participate in mitochondrial biogenesis and modulate a number of transcription factors (e.g., Nrf2), thereby boosting the antioxidant protection system and mitigating tissue injury [61]. Chen et al. [62] mentioned that SIRT-1 deficiency impairs mitochondrial performance, leading to excessive release of ROS. In addition, it has been reported that SIRT-1 is involved in hepatic metabolism through its capacity to deacetylate essential metabolic factors, including PGC-1α, a vital trigger of oxidative stress-combating mechanisms [21,22]. PGC-1α is responsible for the activation of Nrf1 and Nrf2 [63]. Nrf2, a redox sensor protein, is a principal regulator of cytological defense response against oxidative insult, promoting the expression of multiple antioxidant and detoxifying enzymes [64]. Under normal conditions, Nrf2 is trapped by Kelch-like erythroid cell-derived protein 1 (Keap1) in the cytoplasm. Oxidative stress triggers the release of Nrf2, which then moves to the nucleus to induce downstream antioxidant genes, including HO-1 and SOD [65]. In agreement with the obtained outcomes, Ucar et al. [66] recorded a substantial decrease in the mRNA levels of Nrf2 in the hepatic tissues of zebrafish exposed to TMX. To the best of our knowledge, we are the first researchers to elucidate the suppressing effect of TMX on SIRT-1 and PGC-1α genes.

In this investigation, therapy with TAU and/or GA attenuated TMX-induced oxidative insult, as reflected by a marked drop in MDA levels, along with augmented SIRT-1, PGC-1α, Nrf2, and HO-1 transcription levels, increased SIRT-1 and PGC-1α protein concentrations, enhanced SOD and CAT activities, and elevated GSH content in liver tissues. Our findings align with those of Ince et al. [41], who documented that administration of TAU alleviated oxidative stress and restored the activity of antioxidant enzymes in liver tissues of malathion-administered rats; they attributed this ameliorative action of TAU to its potent antioxidant feature. In the same vein, Schaffer et al. [67] ascribed the antioxidant properties and the osmoregulatory action of TAU to its ability to sweep free radicals, thereby mitigating lipid peroxidation. According to de Vries et al. [68], TAU promotes Nrf2, resulting in the activation of the antioxidant system. Moreover, Ouyang et al. [69] reported that TAU strengthened hepatic antioxidant defenses, alleviated oxidative insult, and attenuated hepatic damage in type 2 diabetic rats by triggering Nrf2 antioxidant signaling. Furthermore, GA has been proven to ameliorate hepatic lipid peroxidation and enhance antioxidant status in cyclophosphamide-treated rats [48]. Furthermore, Sanjay et al. [70] found that GA displayed a palliative effect against rifampicin- and isoniazid-induced hepatic impairment by restoring oxidant–antioxidant equilibrium via upregulating Nrf2 signaling. Supporting this, Chang et al. [71] demonstrated that polyphenols, including GA, exhibit an antioxidant activity via stimulating SIRT-1/PGC-1α signaling [71]. It has been reported that GA can induce the SIRT/Nrf2 pathway in HepG2 hepatocellular carcinoma cell lines [72].

Previous studies have established that redox imbalance initiates inflammatory cascades by upregulating NF-κB expression, which subsequently promotes the production of pro-inflammatory cytokines and further exacerbates ROS synthesis [73,74]. In addition to NF-κB, the NLRP3 inflammasome is another free radical sensor implicated in promoting the inflammatory process [75]. Rubartelli [76] and He et al. [77] documented that alterations in redox status trigger NLRP3 expression, stimulating caspase 1 and thereby upregulating IL-1β and IL-18. In addition, NLRP3 has been proven to be stimulated by NF-κB [78]. In this context, our study showed a remarkable increase in the concentrations of NF-κB, Phospho-NF-κB p65, iNOS, IL-1β, mRNA levels of NLRP3, and immunoexpression of TNF-α, along with a drastic drop in the levels of IL-10, an anti-inflammatory marker, in the hepatic tissue of TMX-treated rats. These observations are consistent with those of Abd Elkader et al. [58], who recorded a considerable augmentation in NF-κB, IL-1β, TNF-α, and IL-6 concentrations in the brain tissues of rats administered TMX at 100 mg/kg for 4 weeks. Duzguner and Erdogan [59] reported comparable findings in the liver tissues of rats exposed to IMI.

Notably, the results of this current project demonstrated that supplying TMX-exposed rats with TAU and/or GA alleviated deterioration of inflammatory indices. Similarly, Ince et al. [41] found that TAU therapy ameliorated malathion-induced hepatic inflammation in rats by reducing the transcription levels of TNF-α and IL-1β. The anti-inflammatory effect of TAU has been confirmed in several other mouse and rat liver injury models [79,80]. Moreover, GA has been shown to attenuate liver inflammation caused by carbon tetrachloride through suppressing the release of inflammatory cytokines including cyclooxygenase-2 (Cox-2), IL-1β, IL-6, and TNF-α [53]. In the same vein, Singla et al. [81] reported that the anti-inflammatory action of GA occurs through the repression of the phosphorylation of NF-κB and the inhibitory subunit of NF Kappa B Alpha (IkBa). Additionally, Lin et al. [82] showed that GA exhibited prominent anti-inflammatory effects in gouty arthritis mice by downregulating NLRP3 and IL-1β. The anti-inflammatory effects of TAU and GA in the current research may be attributed to their upregulation of the SIRT-1/PGC-1α pathway. In this context, the crucial role of SIRT-1 in controlling inflammation has been attributed either to its ability to deacetylate NF-κB or to its ability to prevent free radical-induced NF-κB nuclear translocation by enhancing antioxidant defenses [23].

Prior researchers have demonstrated that free radicals and cytokines act synergistically to induce and orchestrate cell apoptosis [60,83]. Accordingly, in this investigation, hepatic Bcl-2 immunoexpression, an anti-apoptotic protein, was downregulated, while the immunoexpression of Bax (a pro-apoptotic protein), along with p53 mRNA and protein levels, as well as caspase-3 and cleaved caspase-3 concentrations, were substantially increased in TMX-subjected rats. Oren [84] reported that p53 and caspase-3 cascade activation is crucial for initiating apoptosis, causing chromatin condensation and cell shrinkage. Overproduction of cytokines and ROS triggers p53 expression, which in turn enhances Bax, with a subsequent reduction in mitochondrial membrane potential, leading to disruption of its permeability [85]. According to Green [86] and Redza et al. [87], Bax and free radicals can cause destruction of mitochondrial DNA and liberation of cytochrome c, which interact with ATP, procaspase-9, and apoptotic protease activating factor-1 (Apaf-1), producing an apoptosome complex in the cytoplasm that stimulates caspase-3, leading to breakdown of cytological proteins, destruction of DNA, additional cytochrome c discharge, and eventually death of cells. Our findings were consistent with those of Abd Elkader et al. [58], who reported that TMX administration in rats induced apoptosis in their brain tissues through elevating the caspase-3 expression. These results are supported by those of Abdelgawad et al. [88], who reported the involvement of the apoptotic pathway in IMI-induced hepatotoxicity in rats.

In the present experiment, TAU and/or GA reversed the TMX-induced surge of p53 mRNA and protein levels, caspase-3 and cleaved caspase-3 concentrations, and Bax immunoexpression, while markedly increasing Bcl-2 expression in hepatic tissues. Similarly, Rashid et al. [89] stated that the administration of TAU resulted in the downregulation of Bax and caspase-3 expression and upregulation of Bcl-2 in the hepatic tissues of rats with alloxan-induced diabetes. Furthermore, GA has been reported to exhibit a gastroprotective effect on ischemia-reperfusion (I/R) injury via its anti-apoptotic action by decreasing the protein expression of caspase-3 [90]. TAU or GA’s anti-apoptotic effects in this experiment may be attributed to the activation of the SIRT-1/PGC-1α pathway. In the same vein, it has been reported that SIRT-1 has the ability to suppress the transcriptional activity of p53 by deacetylating it, thereby inhibiting apoptosis [91].

A limitation of this study is that it did not assess the pharmacokinetic profiles of TAU, GA, and TMX, which could have provided insights into potential interactions among the three compounds and clarified the influence of pharmacokinetic changes on the observed ameliorative effects of TAU and GA against TMX-provoked hepatotoxicity. Future studies are warranted to investigate these possible pharmacokinetic interactions and clarify their contribution to the observed protective effects. Another limitation of the current research is that, although the combination of TAU and GA exhibited enhanced protective efficacy compared to either compound alone, it did not explore the methods that are capable of identifying the underlying mechanisms of this enhancement. Specifically, it remains unclear whether the observed effect is due to an additive or a potentiating interaction. Thus, further advanced investigations are needed to characterize the nature of this interaction more precisely. A third limitation of this study is the lack of in-depth molecular investigations to provide clearer insight into the mechanistic effects of the SIRT1/PGC-1α pathway activation, particularly in relation to mitochondrial rescue and the suppression of TMX-induced inflammation and apoptosis.

## 4. Materials and Methods

### 4.1. Chemicals

Thiamethoxam (TMX, Actara^®^ 25 WG, CAS No.153719-23-4), a product of Syngenta Crop Protection Agrochemicals, Greensboro, NC, USA, was utilized in this study. Gallic acid (GA, CAS No. 149-91-7) and taurine (TAU, CAS No. 107-53-7) were supplied from Sigma Aldrich Co. (St. Louis, MO, USA).

### 4.2. Animals

Forty-two male Wistar albino rats (6–7 weeks old, 160–180 gm) were provided from the animal house, Zagazig University. They were housed in cages at standardized ambient circumstances (23–25 °C temperature, 60 ± 5% humidity, and 12/12 h dark/light shift). Rats were provided with unrestricted supply of food pellets and water and were allowed to acclimate to the surrounding conditions for 7 days prior to the start of the study. The design and procedures of this investigation were reviewed and endorsed by the Mansoura University Animal Care and Use Committee.

### 4.3. Sample Size Calculation

The sample size was determined based on an earlier report by Hamed et al. [45] using G*power 3.1.9.4 software, considering the variation between two independent experimental groups (control vs. TMX groups) adopting a *t*-test with a probability of type I error (α) = 0.05 and a power of 0.80. The sample size was calculated to be 42 rats (6 rats/group).

### 4.4. Experimental Plan

The investigated rats were randomly allocated into 7 groups (*n* = 6 rats). The rats in the control group received normal saline orally (1.5 mL/rat, vehicle control). The TAU group received TAU by gastric gavage (50 mg/kg b.wt./day). The GA group was gavaged with GA at a dose of 20 mg/kg b.wt./day [92,93]. The TMX group received an oral dose of TMX (1/20 LD50 = 78.15 mg/kg b.wt./day) [13,47]. The TMX + TAU group was administered TAU, followed by TMX after 30 min at the same dosages. The TMX + GA group was gavaged with GA 30 min before TMX oral administration. The TMX + TAU + GA group was administered TMX, TAU, and GA in the same manner and at the same doses as previously described. Doses of TAU and GA were selected based on previously published studies [93,94]. All compounds were freshly prepared daily and dissolved individually in 0.5 mL of normal saline. To prevent volume-associated bias, the total provided volume was standardized to 1.5 mL/rat/day in all groups by adjusting the remaining volume with saline when only one or two compounds were administered. The animals received these treatments daily for 4 weeks. Each rat was weighed weekly, and the initial (measured on the 0th day of the study) and final body weights were used to assess the changes in body weight.

### 4.5. Samples Collection

After 24 h of the last dose of treatments (on day 29 of the experiment), all rats were anesthetized using a mixture of ketamine and xylazine i.p. at 50 mg/kg and 5 mg/kg, respectively, to collect blood specimens from their retro-orbital plexus. After centrifuging blood samples at 4000× *g* for 15 min, sera were harvested and preserved at −80 °C for further screening of liver function markers. Afterwards, the animals were decapitated under deep anesthesia, and livers were surgically separated, removed, and weighed. The right lobe of the dissected liver from each rat was divided into two portions: The first portion was homogenized, and the supernatant was isolated by centrifugation for further analysis of redox status indicators. The second portion was maintained at −80 °C for conducting quantitative real-time polymerase chain reaction (qRT-PCR) and enzyme-linked immunosorbent assay (ELISA). Meanwhile, the left lobe of each rat’s liver was immersed in formalin (10%) for microscopic screening.

### 4.6. Biochemical Investigations

#### 4.6.1. Serum Biomarkers of Hepatic Function

The activities of serum alanine aminotransferases (ALT, cat. No. 12212) and alkaline phosphatase (ALP, cat. No. 12117) (Human Diagnostics, Wiesbaden, Germany), as well as the concentrations of total protein (cat. No. 1001291), albumin (cat. No. 1001020) (Spinreact Co., Santa Coloma, Girona, Spain), and total bilirubin (cat. No. BIL099160, BioMed Co., Cairo, Egypt), were assessed employing a spectrophotometer in accordance with the guidelines supplied in the enclosed leaflet.

#### 4.6.2. Determination of Oxidative Stress Indices in Hepatic Tissues

Using commercial kits brought from Biodiagnostic Co. (Dokki, Giza, Egypt), the redox status parameters in hepatic specimens were evaluated following the instructions in the attached booklets. Malondialdehyde (MDA, cat. No. MD 25 29) levels were measured applying the technique of Ohakawa et al. [94]. Also, the concentrations of nitric oxide (NO, cat. No. NO 25 33) were determined based on the protocol of Montgomery and Dymock [95]. Moreover, the activities of superoxide dismutase (SOD, cat. No. SD 25 21) and catalase (CAT, cat. No. CA 25 17), in addition to GSH level (cat. No. GR 25 11), were assessed as illustrated by Nishikimi et al. [96], Aebi [97], and Beutler et al. [98], respectively.

#### 4.6.3. ELISA Analysis

The detection of hepatic levels of caspase-3 (cat. No. E4592–100, Biovision Co., Milpitas, CA, USA), NF-κB (cat. No. ER1186, Wuhan Fine Biotech Co., Wuhan, Hubei, China), iNOS (cat. No. NBP2–80257), p53 (NBP2-75359) (Novus Biologicals Co., Centennial, CO, USA), IL-1β (cat. No. E0119Ra, BT LAB Co., Shanghai, China), and interleukin-10 (IL-10, cat. No. SEA056Ra, Cloud-Clone Corp., Katy, TX, USA), SIRT-1 (cat. No. BSKR65911, Bioss Antibodies Inc., Woburn, MA, USA), PGC-1α (cat. No. SL0828Ra), Phospho-NF-κB p56 (cat. No. SLD1755Ra), and cleaved caspase-3 (cat. No. SL13666Ra) (Sunlong Biotech CO., Hangzhou, Zhejiang, China) was carried out utilizing ELISA kits according to the manufacturer’s directions.

### 4.7. qRT-PCR for the Transcription Levels of SIRT-1, PGC-1α, Nrf2, HO-1, NLRP3, and p53 in Liver Specimens

Trizol reagent (Direct-zol RNA MiniPrep, catalog No. R2050) was implemented in compliance with the supplier’s guidelines to extract total RNA from liver tissues. The isolated RNA’s quality was checked via a NanoDrop (UV–Vis spectrophotometer Q5000, San Jose, CA, USA). The picked RNA was employed to produce cDNA strands by the aid of SensiFastTM cDNA synthesis kit (Bioline Ltd., London, UK, cat. No. Bio-65053). To assess the mRNA levels of SIRT-1, PGC-1α, nuclear factor erythroid 2-related factor (Nrf2), heme oxygenase-1 (HO-1), NLRP3 inflammasome, and p53, a Stratagene MX3005P real-time PCR machine (Agilent, Santa Clara, CA, USA) was operated utilizing SYBR Green PCR Master Mix (2× SensiFastTM SYBR, Bioline Ltd., London, UK) and the primers reported in Table 4. β-Actin, a reference housekeeping gene, was included to standardize the expression of the investigated genes. The following qRT-PCR cycling conditions were applied: 94 °C for 15 min, followed by 40 cycles of 94 °C for 15 s, with primer annealing at 58 °C (30 s) for PGC-1α and at 60 °C (30 s) for the other analyzed genes, and finally at 72 °C (30 s) for extension. Relative assessment of the mRNA levels of the explored genes was accomplished by applying the 2^−ΔΔCt^ approach as shown by Pfaffl [99].

### 4.8. Histopathological Scrutinization of Liver Samples

The liver specimens kept in 10% formalin underwent a classical histological process initiated by dehydration g employing escalating concentrations of alcohol and finalized by submerging in paraffin. Then, using a microtome, the paraffin sections were sliced into 4 µm thick layers that were mounted on glass slides and stained with hematoxylin and eosin (H&E), as delineated by Suvarna et al. [107], to be inspected with an ordinary microscope. Lesions in the liver were graded as demonstrated by Gibson-Corley et al. [108]. To evaluate the lesions, 3 distinct fields were examined in every slide. A blinded manner was utilized for scoring [score scale: 0 = normal; 1 ≤ 25%; 2 = 26–50%; 3 = 51–75%; 4 = 76–100%]. Rat liver lesions were evaluated based on the extent of congestion, inflammation, necrosis of hepatic cells, collagen deposition, and biliary hyperplasia.

In addition, Masson’s trichrome was used to stain hepatic sections to assess the presence and degree of fibrosis. The stained sections were examined using a light microscope. To determine collagen deposition scores, Image J software (1.53n National Institutes of Health, Bethesda, MD, USA) was used.

### 4.9. Immunohistochemistry

Hepatic sections were immunohistochemically stained to investigate the expression of TNF-α, Bax, and B-cell lymphoma 2 (Bcl-2) following the protocols of Chu et al. [109], Jin et al. [110], and Zhao et al. [111]. Briefly, following deparaffinization, the liver sections were immersed in decreasing concentrations of alcohol for rehydration. Thereafter, for antigen retrieval, the sections underwent microwave heating in citrate buffer for 10 min. The slides were then submerged in 3% methanol–H_2_O_2_ for approximately half an hour to block endogenous peroxidase. To prevent non-specific staining, 10% normal blocking serum was applied and left on the slides for 1 h. The slides were then incubated overnight at 4 °C with the primary antibodies against TNF-α (mouse monoclonal IgG2b, 1:100 dilution, cat. No. 60291-1-Ig), Bax (rabbit polyclonal IgG, 1:100 dilution, cat. No. 50599-2-Ig), and Bcl-2 (rabbit polyclonal IgG, 1:100 dilution, cat. No. 26593-1-AP) (Proteintech Group, Inc., Rosemont, IL, USA). After rinsing the slides with PBS, the horseradish peroxidase (HRP)-conjugated anti-mouse and anti-rabbit secondary antibodies were added and maintained on the slidesfor 20 min at 25 °C. Color development was achieved by adding diaminobenzidine (DAB) reagent. A light microscope was utilized to examine the slides after counterstaining with hematoxylin. Image J software (National Institutes of Health, Bethesda, MD, USA) was applied to assess immunohistochemical intensities.

### 4.10. Statistical Analysis

The statistical computation of the findings was performed utilizing GraphPad Prism 5 (GraphPad Software, San Diego, CA, USA). All numerical results were demonstrated as mean ± SEM. To verify the normality of the values, the Shapiro–Wilk approach was applied. The results of the examined groups were compared implementing one-way ANOVA and Tukey’s post hoc analysis. A *p*-value < 0.05 was designated as a statistical significance threshold. ClustVis (https://biit.cs.ut.ee/clustvis/ accessed on 12 June 2025) was used for performing principal component analysis (PCA) and heat map for all data except the protein concentrations measured by ELISA [112]. For heat map analysis, average clustering was conducted with the tightest cluster represented first.

## 5. Conclusions

**Figure 9:**
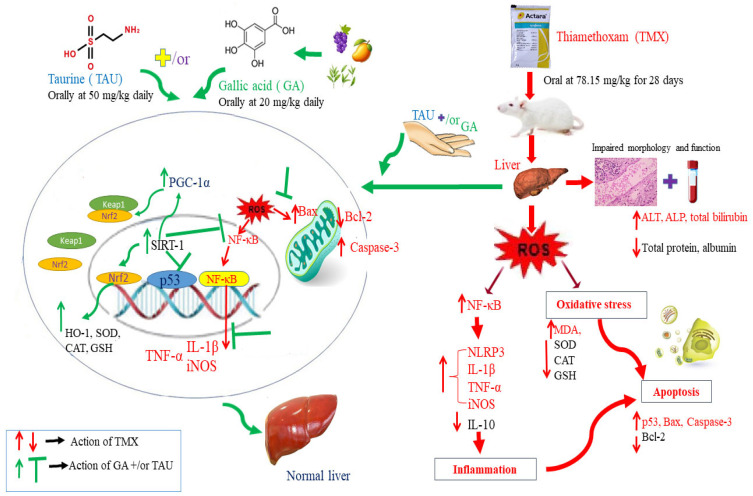
Graphical abstract demonstrating the potential mechanisms of TAU and GA in ameliorating TMX-provoked hepatotoxicity in rats.

The current investigation provides a basis for employing GA and/or TAU as protective agents against hepatic damage caused by TMX. The protective action of these natural products is achieved through correcting the oxidant–antioxidant imbalance, abolishing inflammatory responses, and repressing apoptotic changes through the regulation of SIRT-1/PGC-1α, NF-κB/iNOS, and p53/Bax/caspas-3 signaling pathways. In addition, this study demonstrated a stronger palliative effect of GA compared to TAU against TMX-induced hepatotoxicity. Furthermore, the combination of GA and TAU displayed potentiated hepatoprotective activity, rendering this concurrent remedy a promising approach for alleviating TMX-provoked liver injury. Nevertheless, additional investigations are recommended to fully explore the molecular mechanism mediating the ameliorative effect of GA and TAU against TMX hepatotoxicity and to translate these findings into clinical settings. The suggested mechanisms of TAU and GA in alleviating TMX-provoked hepatotoxicity in rats are illustrated in Figure 9.

## Figures and Tables

**Table 1:** Body weight changes in the study groups.

Experimental Groups	Body Weight (gm)					Body Weight Changes (%)
	0 day	7th day	14th day	21st day	28th day	
Control	172.53 ± 3.03	183.55 ± 3.69	190.26 ± 4.75	196.76 ± 5.40	204.35 ± 7.61	18.25 ± 2.52
TAU	170.60 ± 2.45	182.45 ± 3.15	192.73 ± 3.90	201.45 ±4.47	207.93 ± 8.20	21.70 ± 3.36
GA	171.08 ± 2.91	184.33 ± 4.23	195.43 ± 5.45	205.02 ± 5.13	211.28 ± 9.29	23.24 ± 3.48
TMX	173.32 ± 2.39	176.16 ± 3.44	167.25 ± 5.41 *	161.53 ± 6.11 **	158.10 ± 8.48 **	−9.01 ± 3.69 ***
TMX + TAU	170.16 ± 2.96	173.55 ± 3.03	177.60 ± 3.68	180.73 ± 3.88	184.72 ± 3.53 ^#^	8.53 ± 0.54 ^##^
TMX + GA	174.28 ± 2.41	176.72 ± 2.81	179.25 ± 2.69	187.15 ± 3.43 ^#^	196.43 ± 6.02 ^#^	12.59 ± 2.05 ^###^
TMX + TAU + GA	171.76 ± 2.34	175.81 ± 2.99	183.71 ± 3.65	194.02 ± 4.12 ^##^	201.51 ± 6.32 ^##^	17.18 ± 2.11 ^###^

Values are listed as mean ± SEM (n = 6). One-way ANOVA and Tukey’s post hoc analysis were used for comparing the data between the experimental groups. Significant differences in each column are indicated by superscript symbols (* *p* < 0.05, ** *p* < 0.01 *** *p* < 0.001 vs. the control group; ^#^ *p* < 0.05, ^##^ *p* < 0.01 and ^###^ *p* < 0.001 vs. the TMX group). TAU, taurine; GA, gallic acid; TMX, thiamethoxam.

**Table 2:** Effects of taurine and/or gallic acid on thiamethoxam-induced changes in hepatic performance indices.

Experimental Groups	ALT (U/L)	ALP (U/L)	Total Protein (g/dL)	Albumin (g/dL)	Total Bilirubin (mg/dL)
Control	27.25 ± 2.21	193.42 ± 8.97	8.60 ± 0.35	4.86 ± 0.37	0.25 ± 0.02
TAU	28.18 ± 2.53	191.53 ± 7.27	9.10 ± 0.54	5.08 ± 0.34	0.27 ± 0.03
GA	25.40 ± 2.77	187.26 ± 9.67	8.95 ± 0.41	4.60 ± 0.25	0.22 ± 0.02
TMX	74.56 ± 5.94 ***	372.88 ± 23.49 ***	3.85 ±0.45 ***	1.76 ± 0.19 ***	1.33 ± 0.17 ***
TMX + TAU	48.38 ± 4.62 ** ^###^	264.61 ± 20.96 * ^###^	6.70 ± 0.48 ^##^	3.18 ± 0.27 ** ^#^	0.68 ± 0.04 ** ^###^
TMX + GA	35.85 ± 3.80 ^###^	241.11 ± 16.73 ^###^	7.15 ± 0.57 ^###^	3.61 ± 0.26 ^##^	0.53 ± 0.05 ^###^
TMX + TAU + GA	31.00 ± 3.10 ^### ∆^	215.30 ± 12.36 ^###^	8.01 ± 0.34 ^###^	4.40 ± 0.34 ^###^	0.34 ± 0.03 ^### ∆^

Results are represented as mean ± SEM (n = 6). For data comparison, one-way ANOVA followed by Tukey’s post hoc analysis was used. Significant differences in each column are indicated by superscript symbols (* *p* < 0.05, ** *p* < 0.01, and *** *p* < 0.001 vs. the control group; ^#^ *p* < 0.05, ^##^ *p* < 0.01 and ^###^ *p* < 0.001 vs. the TMX group; and ^∆^ *p* < 0.05 vs. the TMX + TAU group). TAU, taurine; GA, gallic acid; TMX, thiamethoxam; ALT, alanine amino transferase; ALP, alkaline phosphatase.

**Table 3:** Effect of taurine and/or gallic acid on redox status of liver tissues of rats treated with thiamethoxam.

Experimental Groups	MDA (nmole/g Tissue)	NO (µmol/g Tissue)	SOD (U/g Tissue)	CAT (U/g Tissue)	GSH (mg/g Tissue)
**Control**	46.40 ± 4.25	63.18 ± 4.43	42.61 ± 3.60	6.28 ± 0.27	16.98 ± 1.78
**TAU**	43.76 ± 3.79	62.56 ± 4.90	44.10 ± 3.35	6.11 ± 0.43	19.78 ± 1.91
**GA**	38.68 ± 4.46	59.45 ± 3.96	48.21 ± 4.48	6.65 ± 0.35	20.65 ± 1.86
**TMX**	110.32 ± 8.78 ***	142.60 ± 12.17 ***	17.23 ± 1.58 ***	2.13 ± 0.31 ***	4.31 ± 0.59 ***
**TMX + TAU**	81.08 ± 6.31 ** ^#^	103.61 ± 5.01 ** ^##^	29.05 ± 2.45 ^#^	3.83 ± 0.30 *** ^#^	11.46 ± 1.07 ^#^
**TMX + GA**	69.82 ± 3.96 ^###^	87.32 ± 6.44 ^###^	33.08 ± 3.56 ^#^	5.01 ± 0.45 ^###^	14.11 ± 1.15 ^###^
**TMX + TAU + GA**	54.13 ± 5.92 ^### ∆^	76.60 ± 6.75 ^###^	40.15 ± 3.92 ^###^	5.75 ± 0.30 ^### ∆^	16.00 ± 1.63 ^###^

Data are demonstrated as mean ± SEM (n = 6). We utilized one-way ANOVA followed by Tukey’s post hoc test for statistical analysis. Significant differences in each column are indicated by superscript symbols (** *p* < 0.01 and *** *p* < 0.001 vs. the control group; ^#^ *p* < 0.05, ^##^ *p* < 0.01, and ^###^ *p* < 0.001 vs. the TMX group; and ^∆^ *p* < 0.05 vs. the TMX + TAU group). TAU, taurine; GA, gallic acid; TMX, thiamethoxam; MDA, malondialdehyde; NO, nitric oxide; SOD, superoxide dismutase; CAT, catalase; GSH, reduced glutathione.

**Table 4:** The sequences of primers employed for quantitative real-time PCR assay.

Target Gene	Forward Primer (5′-3′)	Reverse Primer (5′-3′)	References
SIRT-1	CACCAGAAAGAACTTCACCACCAG	ACCATCAAGCCGCCTACTAATCTG	Braidy et al. [100]
PGC-1α	AATGAATGCAGCGGTCTTAG	GTCTTTGTGGCTTTTGCTGT	Belviranlı and Okudan, [101]
Nrf2	CACATCCAGACAGACACCAGT	CTACAAATGGGAATGTCTCTGC	Yamashita et al. [102]
HO-1	GGCTTTAAGCTGGTGATGGC	GGGTTCTGCTTGTTTCGCTC	Chu et al. [103]
NLRP3 p53	CAGACCTCCAAGACCACGACTG GTCGGCTCCGACTATACCACTAT	CATCCGCAGCCAATGAACAGAG CTCTCTTTGCACTCCCTGGGGG	Samra et al. [104] Deng et al. [105]
β-actin	TCCTCCTGAGCGCAAGTACTCT	GCTCAGTAACAGTCCGCCTAGA	Banni et al. [106]

## Data Availability

The original contributions presented in the study are included in the article and Supplementary Material, further inquiries can be directed to the corresponding author.

## Supplementary Materials

The following supporting information can be downloaded at: https://www.mdpi.com/article/10.3390/ph18081112/s1, Additional supporting information can be found in the Supplementary Materials including Table S1: Liver Morphology and Hepatosomatic Parameters in the Experimental groups.

## Abbreviations

ALP	Alkaline phosphatase
ALT	Alanine aminotransferases
Bax	Bcl-2-associated X protein
Bcl-2	B-cell lymphoma 2
CAT	Catalase
GA	Gallic acid
GSH	Reduced glutathione
HO-1	Heme oxygenase-1-
IL-1β	Interleukin-1β
IL-10	Interleukin-6
iNOS	Inducible nitric oxide synthase
MDA	Malondialdehyde
MnSOD	Manganese superoxide dismutase
nAChRs	Nicotinic acetyl choline receptors
NF-κB	Nuclear factor kappa
NO	Nitric oxide
NLRP3	Nucleotide-binding domain, leucine-rich-containing family, pyrin domain–containing-3
Nrf2	Nuclear factor erythroid 2-related factor
PGC-1α	Peroxisome proliferator-activated receptor (PPAR) gamma coactivator 1 alpha
SIRT1	Sirtuin-1
TAU	Taurine
TMX	Thiamethoxam
TNF-α	Tumor necrosis factor alpha
WHO	World Health Organization
