# Supplementary material for: Gallic Acid and Taurine Attenuate Thiamethoxam-Induced Hepatotoxicity in Rats by Modulating SIRT-1/PGC-1α, NF-κB/iNOS, and p53/Bax/Caspase-3 Pathways
Source: Pharmaceuticals (Basel). 2025 Jul 25;18(8):1112. doi: 10.3390/ph18081112 (PMC12389281; doi:10.3390/ph18081112)
Supplement: Supplementary file 1 [file pharmaceuticals-18-01112-s001.zip › pharmaceuticals-3729100-supplementary.pdf]

Supplemtary Table S1: Liver Morphology and Hepatosomatic Parameters in the Experimental Groups

**Experimental groups**	**Color**	**Size**	**Visible congestion**	**Liver weight (gm)**	**Hepatosomatic index (%)**
Control	reddish brown	normal	Abscent	7.23 ± 0.18	3.54 ± 0.07
TAU	reddish brown	normal	Abscent	7.30 ± 0.23	3.51 ± 0.09
GA	reddish brown	normal	Abscent	7.11 ± 0.26	3.36 ± 0.12
TMX	pale	slightly enlarged	Marked congetsion	8.35 ± 0.24	5.31 ± 0.25***
TMX + TAU	slightly pale	mild enlargment	Mild congestion	7.8 ± 0.28	4.22 ± 0.17$^{\#\#}$
TMX + GA	near reddish brown	near normal	Mild congestion	7.58 ± 0.31	3.87 ± 0.21$^{\#\#\#}$
TMX + TAU + GA	reddish brown	normal	Minimal to abscent	7.42 ± 0.32	3.70 ± 0.25$^{\#\#\#}$

Results for liver weight and hepatosomatic index are represented as the mean ± SEM (n = 6). For data comparison, one-way ANOVA and Tukey's post-hoc analysis were used. Significant differences in each column were indicated by superscript symbols ($^{***}p < 0.001$ vs. the control group, $^{\#\#}$ $p < 0.01$ and $^{\#\#\#}$ $p < 0.001$ vs. the TMX group .TAU; taurine, GA; gallic acid, TMX; thiamethoxam,
